# No Differences in Wound Healing and Scar Formation Were Observed in Patients With Different COVID-19 Vaccination Intervals

**DOI:** 10.3389/fpubh.2022.883113

**Published:** 2022-06-01

**Authors:** Chen Dong, Zhou Yu, Xin Quan, Siming Wei, Jiayang Wang, Xianjie Ma

**Affiliations:** Department of Plastic Surgery, Xijing Hospital, Fourth Military Medical University, Xi'an, China

**Keywords:** vaccine hesitancy, COVID-19, wound healing, scar formation, vaccination, COVID-19 vaccine, plastic surgery

## Abstract

**Background:**

Safety concerns are one of the most common reasons for COVID-19 vaccination refusal. In the field of plastic and reconstructive surgery, whether COVID-19 vaccination influences wound healing and scar formation is worthy of special attention.

**Methods:**

In this study, patients with adult trauma with subcutaneous sutures placed by a single plastic surgeon in a single center were included. The vaccination interval was defined as the interval between the last dose of the COVID-19 vaccine and when surgical sutures were introduced. The patients were categorized by vaccination interval into three groups of <1, 1–3, and ≥3 months. Wound healing and scar formation were rated according to the Wound Assessment Inventory (WAI) and Patient and Observer Scar Assessment Scale (POSAS) in the groups at 7 days and after a 3-month follow-up.

**Results:**

All total and individual scores of WAI and POSAS were not significantly different among the groups.

**Conclusion:**

No differences in wound healing and scar formation were observed in patients with different COVID-19 vaccination intervals. Thus, it is not necessary to postpone COVID-19 vaccination, as the vaccine does not affect wound healing and scar formation in patients undergoing surgery. This study aimed to eliminate concerns and hesitancy in receiving the COVID-19 vaccine.

## Introduction

Vaccines designed to elicit protective immune responses remain key for containing the COVID-19 pandemic caused by severe acute respiratory syndrome coronavirus 2 (SARS-CoV-2) (1). However, global surveys have revealed that ~30% of participants were hesitant about COVID-19 vaccination (2, 3). Doctors also lack adequate evidence to address vaccine hesitancy, and many doctors are vaccine-hesitant themselves (4, 5). Hesitancy is primarily driven by vaccine safety concerns (6). Although the overall safety of COVID-19 vaccines has been demonstrated by placebo-controlled trials (7), few studies on whether a specific physiological state or pathological process is changed after the COVID-19 vaccination have been published (8–10).

Research on wound healing and scar formation is highly valued by plastic surgeons (11, 12). In our daily clinical practice, concerns about vaccine safety are manifested in the thought that vaccination may be detrimental to wound healing and result in scar formation after surgery, which is a common concern of patients we have treated during the pandemic. Until now, no evidence-based study has been published regarding how soon patients can undergo plastic and aesthetic surgery after receiving the COVID-19 vaccine and whether the COVID-19 vaccine affects wound healing and scar formation. Therefore, in this study, differences in wound healing and scar formation were investigated in patients with trauma with subcutaneous sutures after different COVID-19 vaccination intervals.

## Materials and Methods

### Study Design

This study was performed in accordance with the ethical standards of our institution and the 1964 Declaration of Helsinki. This was a retrospective study performed on a consecutive cohort from June 2021 to October 2021 in a single center. Inclusion criteria included patients who (1) were 18–60 years of age, (2) were diagnosed with simple and open skin injuries, who received a full course of COVID-19 vaccination, and (3) underwent subcutaneous suture placement by a single plastic surgeon (CD). Exclusion criteria included patients who (1) were vaccinated after suture placement or (2) were lost to follow-up. The vaccination interval was defined as an interval between the last dose of the COVID-19 vaccine and the surgical suture placement. Patients were categorized by vaccination interval into three groups: (1) <1, (2) ≥1 and <3, and (3) ≥3 months according to the appearance of vaccine side effects and changes in neutralizing antibodies. Most cutaneous reactions after COVID-19 vaccination lasted no more than 30 days (13). At the 3–6-month interval, the level of neutralizing antibodies against COVID-19 plateaued and gradually decreased (14, 15). Surgical wound healing of the patients was assessed according to the Wound Assessment Inventory (WAI) at 7 days. The WAI has good validity and was designed to visually judge the apparent degree of soft tissue healing in post-surgical incision wounds according to three criteria: edema, erythema, and exudates (16). Scar formation was evaluated according to the Patient and Observer Scar Assessment Scale (POSAS) after a 3-month follow-up. POSAS is a reliable and feasible tool for scar assessment that includes both a patient and an observer scar assessment scale (17). Moreover, vaccination time, doses, and type of COVID-19 vaccine were recorded preoperatively and at the 3-month follow-up. The main outcomes were the scale scores of wound healing and scar formation. Other outcomes were complications during the 3-month follow-up, such as surgical site infection and wound dehiscence, among others.

### Bias Control

#### Selection Bias

- The cohort was consecutive during the COVID-19 pandemic.
- One surgeon performed the surgeries, which avoided the bias of different surgical techniques.
- The vaccination interval in the study was almost random because the wound sutures were unplanned surgeries, which reduced patients' and surgeons' subjective selection bias.

#### Information Bias

- All ratings were given independently by two plastic surgeons (XQ and SW) and were analyzed by a third person (JW).
- Clinical images were obtained after patient consent after verification by a senior author (ZY, not publicly available).

#### Confounding Bias

- All patients were diagnosed with simple and open skin injuries, which eliminated interference with the results by other comorbidities.
- Subgroup analyses were conducted to evaluate the effects of different COVID-19 vaccine types.

### Statistical Analysis

The sample size was estimated using the following formula (18):


$$\begin{array}{l} n=2\left(\sigma \frac{z_{1-\alpha /\left(2\tau \right)+z_{1-\beta }}}{\mu_{A}-\mu_{B}}\right) \end{array}$$

According to the previous publication and clinical observations, the average scores on the POSAS patient scale in groups of <1, ≥1 and <3, and ≥3 months were estimated to be 30, 28, and 20, respectively (19). Also, the standard deviation (SD) of each group was 5. τ= 2, α = 0.05, and β = 0.2. Thus, 8 patients in each group and a total of 24 patients were needed at least. To account for 25% of dropouts, at least 30 patients were needed to recruit for this study. The distribution of data in this study was shown as median (interquartile range). Differences in continuous data and ranked data were evaluated by the Kruskal–Wallis test, and categorical data were evaluated by Fisher's exact test. Values of *p* < 0.05 were considered statistically significant. Analyses were conducted using SPSS Version 25 (IBM, Chicago, IL, USA) and GraphPad Prism Version 7.00 (GraphPad Prism Inc., San Diego, CA, USA).

## Results

### Study Cohort

**Figure 1:**
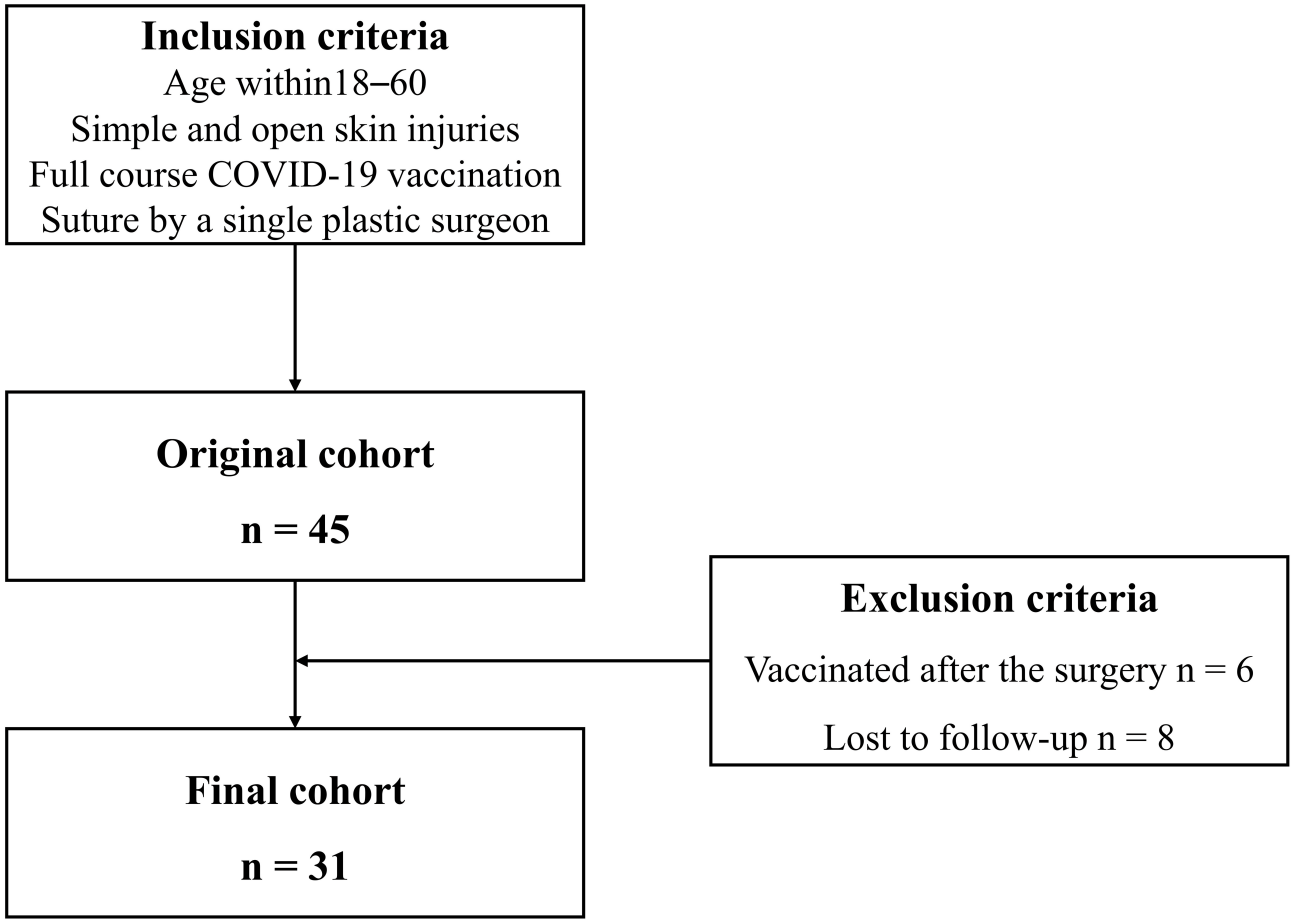
Flow diagram of this study.

A total of thirty-one patients were included in the final cohort. The process of study inclusion is illustrated in the flow diagram in Figure 1. Details of patients' characteristics were shown in Table 1. None of the patient characteristics was statistically different among the three groups [ <1 month (*n* = 8), 1–3 months (*n* = 12), and ≥3 months (*n* = 11)] in age, wound causes, wound sites, wound type, wound length, topical silicone application, and laser therapy. However, in vaccine type, the proportions of inactivated vaccine in the three groups were 62.5, 100, and 54.5%, respectively (*p* =.027).

**Table 1:** Patient characteristics.

	**Group, media (IQR) or** ***n*** **(%)**				
**Items**	** <1 month** **(*n* = 8)**	**1–3 months** **(*n* = 12)**	**≥3 months** **(*n* = 11)**	**Total**	***p***
Age, year	24 (11)	25 (11)	31(11)	26 (11)	0.261*
**Gender**					
Male	5 (62.5)	9 (75.0)	6 (54.5)	20 (64.5)	0.576^#^
Female	3 (37.5)	3 (25.0)	5 (45.5)	11 (35.5)	
**Wound causes**					
Fallen	5 (62.5)	4 (33.3)	8 (72.7)	17 (54.8)	0.526^#^
Cut	1 (12.5)	4 (33.3)	1 (9.1)	6 (19.4)	
Smashed	2 (25.0)	3 (25.0)	1 (9.1)	6 (19.4)	
Bitten	–	1 (8.3)	1 (9.1)	2 (6.5)	
**Wound sites**					
Head & face	7 (87.5)	10 (83.3)	10 (90.9)	27 (87.1)	0.545^#^
Trunk	1 (12.5)	–	–	1 (3.2)	
sLimbs	–	2 (16.7)	1 (9.1)	3 (9.7)	
**Wound type**					
Lacerations	8 (100.0)	8 (66.7)	10 (90.9)	26 (83.9)	0.201^#^
Avulsions	–	3 (25.0)	–	3 (9.7)	
Defects	–	1 (8.3)	1 (9.1)	2 (6.5)	
Wound length, cm	4 (2)	4 (2)	3 (1)	4 (2)	0.851*
Interval from injury to surgery, hr	12 (9)	14 (10)	16 (23)	14 (11)	0.369*
Surgical interval, min	35 (28)	53 (23)	45 (20)	45 (25)	0.122*
**Topical silicone application**					
No	3 (37.5)	5 (41.7)	4 (36.4)	12 (38.7)	1.000^#^
Yes	5 (62.5)	7 (58.3)	7 (63.3)	19 (61.3)	
**Laser therapy**					
No	8 (100.0)	12 (100.0)	10 (90.9)	30 (96.8)	0.613^#^
Yes	–	–	1 (9.1)	1 (3.2)	
**Vaccine type**					
Inactivated	5 (62.5)	12 (100.0)	6 (54.5)	23 (74.2)	0.027^#^
Adenovirus type 5 vector	2 (25.0)	–	4 (36.4)	6 (19.4)	
Others	1 (12.5)	–	1 (9.1)	1 (6.4)	

*Fisher's exact test;

*^#^Krusal-Wallis test; IQR, interquartile range*.

### Primary and Secondary Outcomes

**Figure 2:**
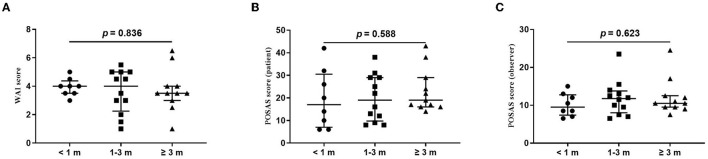
Comparison of total score of wound assessment inventory (WAI) and patient and observer scar assessment scale (POSAS) between patients undergoing the surgical suture with different vaccination intervals. **(A)** WAI at 7 d follow-up; **(B)** POSAS patient scale at three-month follow-up; **(C)**. POSAS observer scale at three-month follow-up; vaccination interval was defined as an interval between the time of the last dose of COVID-19 vaccination and the time of surgical sutures. The numbers of patients in groups of <1 month, 1–3 months, and ≥3 months were 8, 11, and 12, respectively.

**Figure 3:**
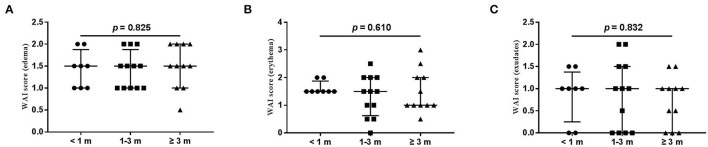
Each item of WAI. **(A)** edema; **(B)** erythema; **(C)** exudates. Numbers of patients in groups of <1 month, 1–3 months, and ≥3 months were 8, 11, and 12, respectively.

**Figure 4:**
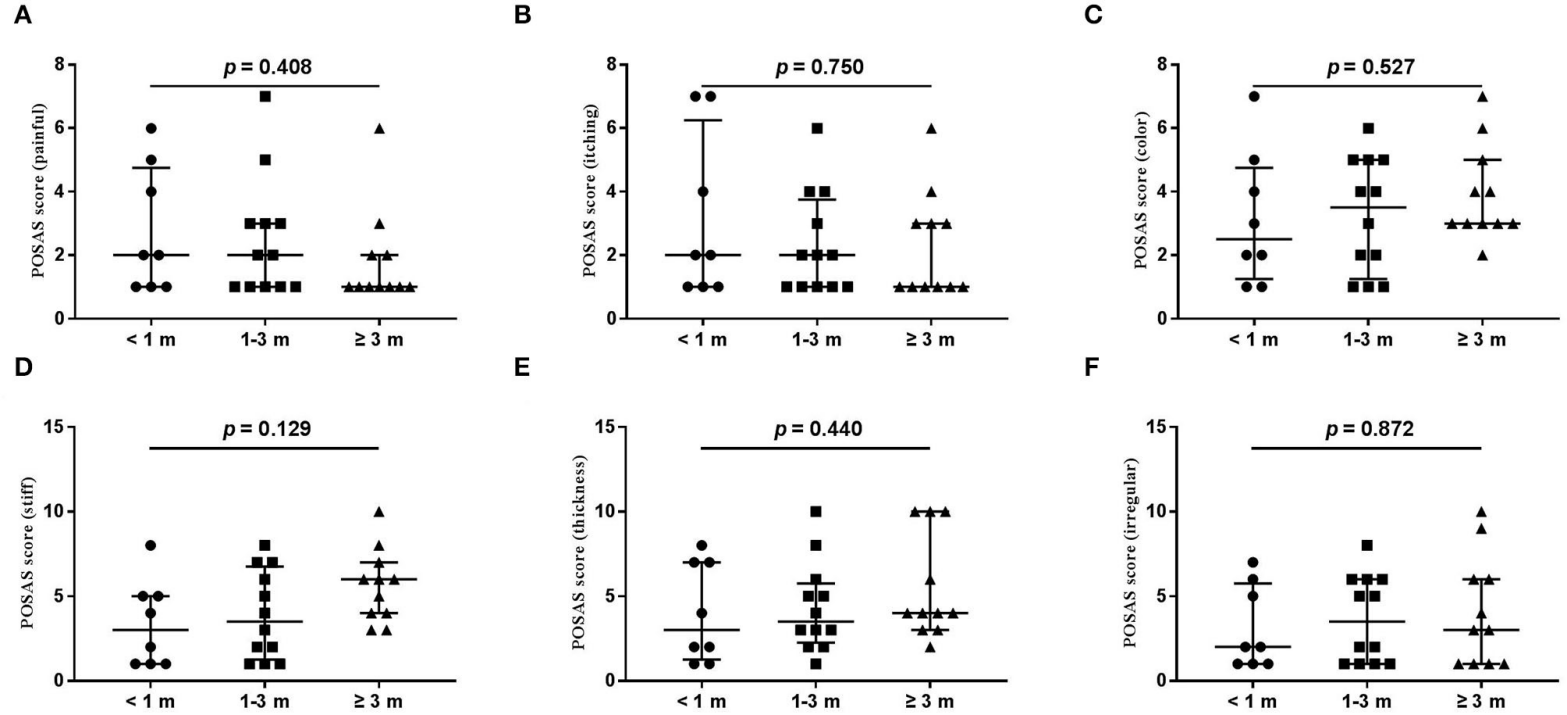
Each item of the POSAS patient scale. **(A)** painful; **(B)** itching; **(C)** color; **(D)** stiff; **(E)** thickness; **(F)** irregular. The numbers of patients in groups of <1 month, 1–3 months, and ≥3 months were 8, 11, and 12, respectively.

**Figure 5:**
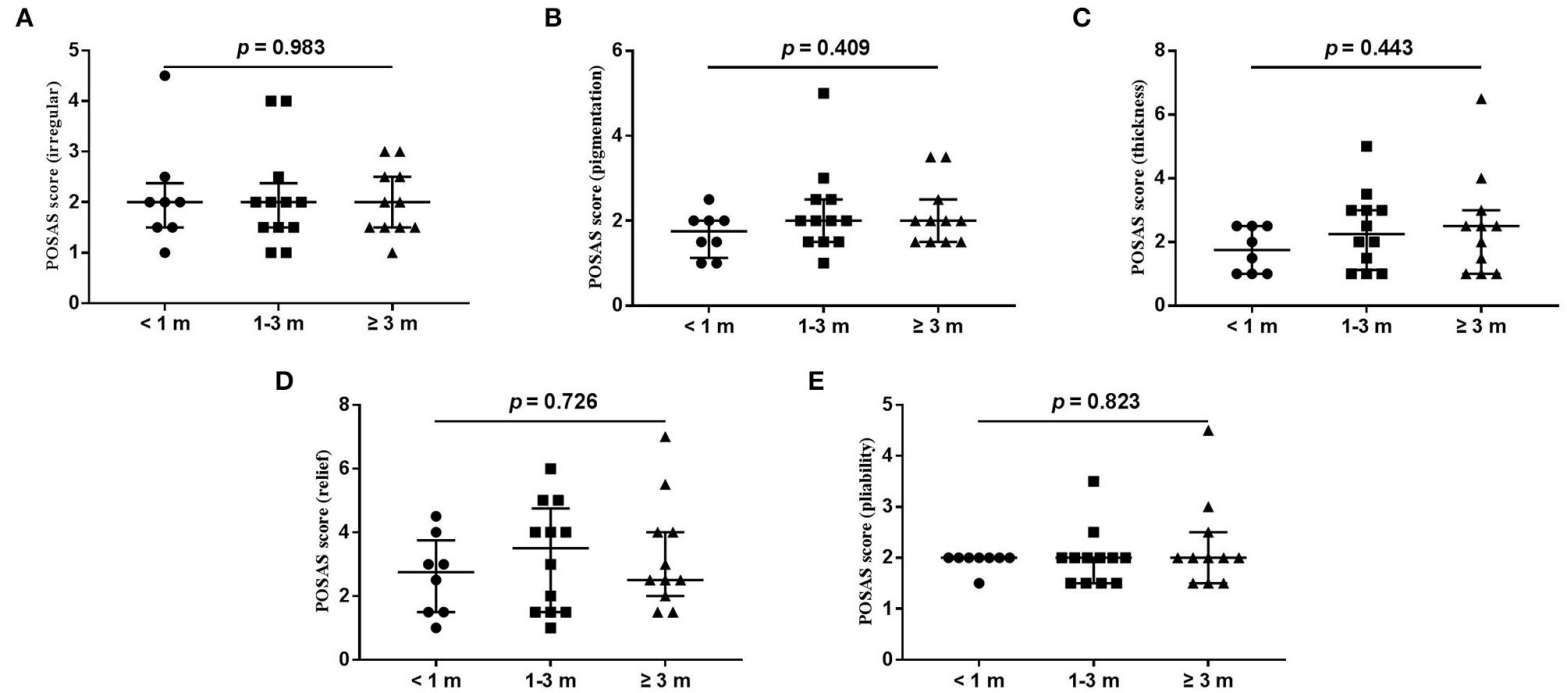
Each item of POSAS observer scale. **(A)** vascularization; **(B)** pigmentation; **(C)** thickness; **(D)** relief; **(E)** pliability. Numbers of patients in groups of <1 month, 1–3 months, and ≥3 months were 8, 11, and 12, respectively.

The wound healing and scar formation assessments by the WAI and POSAS are illustrated in Figure 2. The results of each item for the WAI and POSAS scales are illustrated in Figures 3–5. All total and individual scores of the WAI and POSAS scales showed no statistically significant difference among the groups. No complications were observed in any patients.

### Subgroup Analysis of Different Vaccine Types

In patients who received inactivated vaccine, no statistically significant difference was observed both in wound healing and scar formation among the three groups of <1, 1–3, and ≥3 months (WAI: *p* = 0.553; POSAS patient scale: *p* = 0.399; POSAS observer scale: *p* = 0.976). In patients who received adenovirus type 5 vector vaccine, no statistical difference was observed in wound healing or scar formation between the <1-month group and the ≥3-month group (WAI: *p* = 1.000; POSAS patient scale: *p* = 1.000; POSAS observer scale: *p* = 0.533).

## Discussion

The World Health Organization (WHO) has stated that “vaccine hesitancy” is one of 10 current global health threats (20). Safety concerns are one of the most common reasons for COVID-19 vaccine refusal (21). In the field of plastic and reconstructive surgery, whether COVID-19 vaccination influences wound healing and scar formation is worthy of special attention.

Dermatologic side effects and cutaneous reactions, such as local injection site reactions, morbilliform rash, pernio, pityriasis rosea, and erythema multiforme, due to the COVID-19 vaccine are very common (22). Moreover, cutaneous small-vessel vasculitis after COVID-19 vaccination has also been reported, which may aggravate these existing cutaneous injuries (23, 24). However, after comparing different vaccination intervals, no difference was found in wound healing. This is likely due to a short period, during which cutaneous reactions caused by COVID-19 vaccination occur. McMahon et al. found that local injection site reactions occurred after a median of 1 day and that delayed large local reactions occurred after a median of 7 days after vaccination (13). Wrafter et al. recommended that patients with burn injuries should be vaccinated against SARS-CoV-2 once they recovered from the acute phase of injury (25). Therefore, it is not necessary to postpone COVID-19 vaccination, as the vaccine does not affect wound healing.

Several studies have reported that Bacillus Calmette-Guérin (BCG) local scars are reactivated as a result of the COVID-19 vaccination (26–28). The interaction between angiotensin-converting enzyme 2 (ACE2) receptors and spike proteins of SARS-CoV-2 in the dermis favors a pro-inflammatory, loco-regional TH1 cascade, which promotes a CD8^+^T cell-mediated reaction to incipient granulomas (29). However, no difference in scar formation among different vaccination interval groups was observed in this study. One possible reason is that the patients with scar formation are only isolated cases. Another possible reason is that the reactivation of BCG scars is attributed to vaccine-induced immune activation under T cell bystander stimulation, whereas scars caused by trauma do not exhibit a similar phenomenon (28). Besides, some viruses, such as human T-cell lymphotropic virus type 1 (HTLV-1) and human papillomavirus (HPV), can result in healing dysregulation and infective dermatitis (1, 30). Meanwhile, the COVID-19 vaccine is a type of virus vaccine. The public may be concerned that COVID-19 vaccination will cause side effects similar to viral infections mentioned above to affect wound healing and even lead to hypertrophic scar formation. However, no change in wound healing is observed in our study, possibly attributing to the fact that inactivated vaccines are the main vaccine type used in the Chinese mainland, and the immune mechanism of inactivated vaccines is the stimulation of non-pathogenic viral proteins to the immune system; this may minimize the influence of virus to the participants or patients.

Given the measures of radical debridement, necrotic tissue removal, and fine suturing, primary healing of the wounds was achieved for all patients in this study. Thus, any differences in complication rates were not compared among the groups.

This study has some limitations. First, the follow-up to determine scar formation ended at 3 months because of the widespread prevalence of booster doses on the Chinese mainland. If patients were vaccinated both pre- and post-operatively, the researchers would not have known exactly which dose affected the patients. However, this article does provide preliminary clues in the comparison of the effects of different COVID-19 vaccination intervals on early-stage wound healing and scar formation. Second, the sample size is relatively small. However, all surgeries were performed by the same plastic surgeon, which enhanced comparability among the groups. Third, because the patients in this study came from a single center and were treated by a single surgeon, the conclusions may not be applicable to patients in other centers and treated by other surgeons. Fourth, this is a descriptive study, some basic conditions of patients, such as wound type, have considerable heterogeneity.

## Conclusions

No differences in wound healing and scar formation were observed in patients with different COVID-19 vaccination intervals. Therefore, it is unnecessary to postpone COVID-19 vaccination in patients undergoing surgery if they are concerned that the vaccine affects wound healing and scar formation. This study is beneficial for eliminating concerns and hesitancy regarding COVID-19 vaccines.

## Data Availability Statement

The raw data supporting the conclusions of this article will be made available by the authors, without undue reservation.

## Ethics Statement

The studies involving human participants were reviewed and approved by Fourth Military Medical University. The patients/participants provided their written informed consent to participate in this study.

## Author Contributions

CD: conducting the surgeries, designing the study, acquiring data, and writing the manuscript. ZY: concept of the study, designing the study, acquiring data, and writing the manuscript. XQ and SW: evaluating the scales and editing the manuscript. JW: analyzing data and editing the manuscript. XM: concept of the study, designing experiments, and writing and editing the manuscript. All authors contributed to the article and approved the submitted version.

## Conflict of Interest

The authors declare that the research was conducted in the absence of any commercial or financial relationships that could be construed as a potential conflict of interest.

## Publisher's Note

All claims expressed in this article are solely those of the authors and do not necessarily represent those of their affiliated organizations, or those of the publisher, the editors and the reviewers. Any product that may be evaluated in this article, or claim that may be made by its manufacturer, is not guaranteed or endorsed by the publisher.
